# Supplementary figures and images for: Denoising diffusion weighted imaging data using convolutional neural networks
Source: PLoS One. 2022 Sep 15;17(9):e0274396. doi: 10.1371/journal.pone.0274396 (PMC9477507; doi:10.1371/journal.pone.0274396)

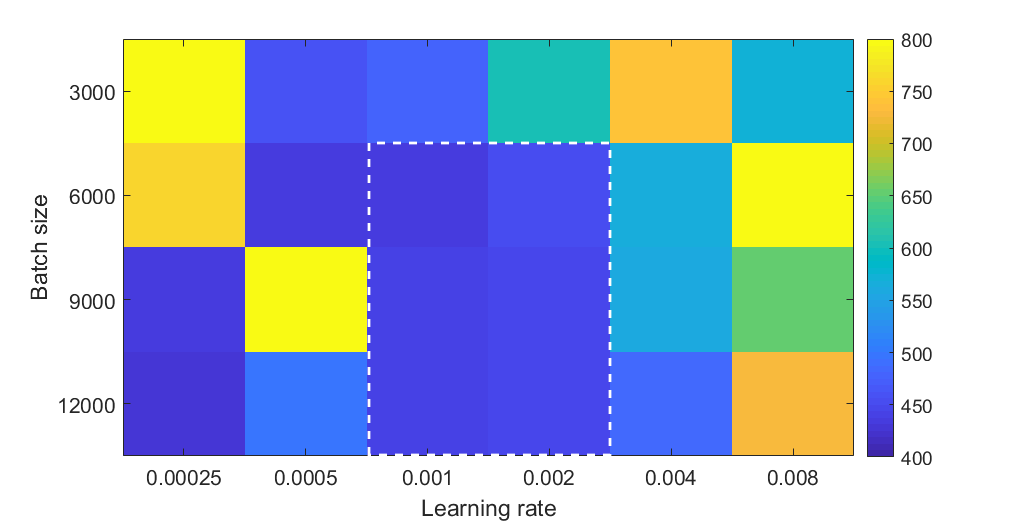

Supplement: S1 Fig — Taking 75% voxels as training set, and the remaining 25% as validation set. After 20000 iterations, we used root-mean-square error as the measure of performance to select the optimal combination of batch size and learning rate. The area outlined by the dashed white line are optimized combinations of batch size and learning rate. We selected a batch size of 6000 and a learning rate of 0.001 for our analyses. (TIF) [file pone.0274396.s001.tif]

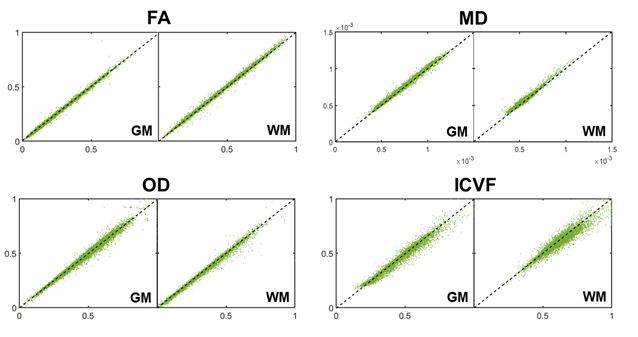

Supplement: S2 Fig — (PNG) [file pone.0274396.s002.png]
